# Migration and invasion is inhibited by silencing ROR1 and ROR2 in chemoresistant ovarian cancer

**DOI:** 10.1038/oncsis.2016.32

**Published:** 2016-05-30

**Authors:** C E Henry, E Llamosas, A Djordjevic, N F Hacker, C E Ford

**Affiliations:** 1 Adult Cancer Program, Level 2, Metastasis Research Group, Lowy Cancer Research Centre and School of Women's and Children's Health, Faculty of Medicine, University of New South Wales, Sydney, NSW, Australia; 2 Gynaecological Cancer Centre, Royal Hospital for Women, Sydney and School of Women's and Children's Health, Faculty of Medicine, University of New South Wales, Sydney, NSW, Australia

## Abstract

Ovarian cancer survival remains poor despite recent advances in our understanding of genetic profiles. Unfortunately, the majority of ovarian cancer patients have recurrent disease after chemotherapy and lack other treatment options. Wnt signalling has been extensively implicated in cancer progression and chemoresistance. Therefore, we investigated the previously described Wnt receptors ROR1 and ROR2 as regulators of epithelial-to-mesenchymal transition (EMT) in a clinically relevant cell line model. The parental A2780- and cisplatin-resistant A2780-cis cell lines were used as a model of ovarian cancer chemoresistance. Proliferation, adhesion, migration and invasion were measured after transient overexpression of ROR1 and ROR2 in the parental A2780 cell line, and silencing of ROR1 and ROR2 in the A2780-cis cell line. Here we show that ROR1 and ROR2 expression is increased in A2780-cis cells, alongside β-catenin-independent Wnt targets. Knockdown of ROR1 and ROR2 significantly inhibited cell migration and invasion and simultaneous knockdown of ROR1 and ROR2 significantly sensitised cells to cisplatin, whilereas ROR overexpression in the parental cell line increased cell invasion. Therefore, ROR1 and ROR2 have the potential as novel drug targets in metastatic and recurrent ovarian cancer patients.

## Introduction

The low survival rates for ovarian cancer patients are due, in part, to the late diagnosis and the development of resistance to traditional chemotherapy in recurrent disease. Despite seminal studies in recent years investigating the genetic and molecular aetiology of epithelial ovarian cancer, particularly the high-grade ovarian cancer serous subtype,^1, 2, 3^ our understanding of ovarian cancer progression and chemoresistance is still very limited.

Used for over 30 years, cisplatin remains a common chemotherapy treatment option following cytoreductive surgery for patients with advanced ovarian cancer.^4^ Cisplatin is a platinum analogue that prevents normal DNA function through reactions that cause intrastrand or interstand crosslinks.^5^ Patients with ovarian cancer generally respond well to chemotherapy but the tumours often recur, which is a clinical challenge.

Ovarian cancer is a heterogeneous disease and each subtype has different propensities to metastasise. Extensive genomic analysis of high-grade ovarian cancer serous subtype has allowed for the classification of four major molecular subtypes: mesenchymal, immunoreactive, differentiated and proliferative.^1, 6, 7, 8, 9^ The mesenchymal phenotype exhibits a shorter disease-specific survival and increased Wnt signalling.^9^ In individual patient case studies, a switch to this mesenchymal phenotype has been shown in samples taken from metastatic sites, such as omental implants in comparison with primary tumour.^7^ Epithelial-to-mesenchymal transition (EMT) has also been implicated in high-grade ovarian cancer serous subtype invasiveness and chemoresistance through *in vivo* studies.^10, 11, 12, 13^ The EMT profile of ovarian cancer cell lines can predict responses to cisplatin, which suggests that the more aggressive, mesenchymal-type cells are more resistant to chemotherapy.^13^

An important signalling cascade involved in EMT regulation is the Wnt signalling pathway, with increasing evidence suggesting that β-catenin-independent signalling has a critical role in this dynamic process.^10, 14, 15, 16, 17, 18, 19, 20, 21, 22^

Recent studies demonstrate that the novel Wnt receptors ROR1 and ROR2 correlate with worse prognosis and drive EMT in a variety of tumour types including breast cancer, cervical cancer and melanoma.^15, 23, 24, 25, 26^ Our laboratory reported that upregulation of Wnt5a in epithelial ovarian cancer regulates EMT^10^ and confirmed that the novel Wnt5a receptor, ROR2, is also upregulated in a patient cohort of ovarian cancer. We recently demonstrated that ROR2 and its sister receptor, ROR1, regulate ovarian cancer migration and invasion.^27^

Patients who develop recurrent and chemoresistant disease generally do not have additional surgery. Therefore, owing to the challenge of patient sample collection, we used a well-established clinically relevant cell line model for continuing the investigation into RORs in ovarian cancer. Here we report that ROR1 and ROR2 expression is increased in this chemoresistant cell line and support our initial findings that ROR1 and ROR2 regulate ovarian cancer cell migration and invasion. ROR1 and ROR2 present as possible targets for novel therapies for the treatment of ovarian cancer.

## Results

### ROR1 and ROR2 expression is increased in the A2780-cis chemoresistant cell line model

Our previous study^27^ identified that ROR1 and ROR2 are upregulated in epithelial ovarian cancer and regulate cell migration and invasion. Clinically, ovarian cancer patients present with aggressive disease, which often recurs after chemotherapy or radiation therapy.^28^ Therefore, we chose to continue our investigation of RORs in epithelial ovarian cancer using a well-established chemoresistant cell line, the A2780-cis model.

A2780-cis cells have an increased expression of both ROR1 and ROR2 at the transcriptional level compared with the parental A2780 cell line (Supplementary Figure 1A). The A2780 cell line had no mRNA expression of ROR2; however, both ROR1 and ROR2 protein were present in each cell line (Supplementary Figure 1B).

In addition to overexpression of both ROR receptors, the A2780-cis cells had increased β-catenin-independent Wnt signalling, as seen by significant upregulation of downstream targets *CJUN*, *RHOA* and *NFAT* (Supplementary Figures 1C; *P*<0.05), and a possible decrease in β-catenin-dependent signalling, as measured by a decrease in *AXIN2* (Supplementary Figure 1D).

Previously, it has been shown that the A2780-cis cells exhibit a mesenchymal phenotype with increased migration and invasion capacity compared with the parental A2780 cells.^11^ Therefore, we confirmed the EMT profile of the A2780-cis cells and similarly found significant upregulation of important EMT genes such as *MMP3* (600-fold increase) and *BMP2* (50-fold increase) (Supplementary Figure 1E) and significant downregulation of *CDH1* (50-fold decrease) and *CDH2* (9000-fold decrease) (Supplementary Figure 1F).

### ROR2, but not ROR1, silencing in A2780-cis decreases proliferation and has no effect on cell adhesion

**Figure 1:**
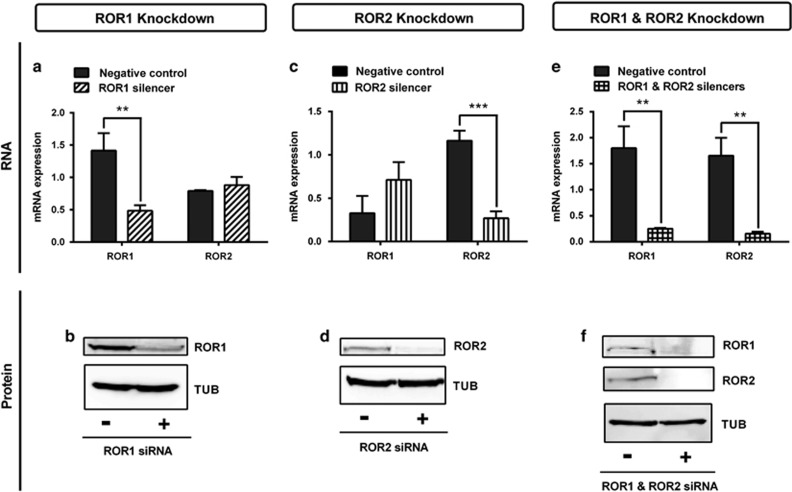
ROR1 and ROR2 silencing using siRNA. (**a**) ROR1 is decreased at the mRNA level following siRNA-induced knockdown in cisplatin serous ovarian cancer (A2780-cis) cells. No effect on ROR2 mRNA level. qRT–PCR was performed in triplicate and normalised to three different housekeeping genes (*SDHA*, *HSPCB* and *RPL13A*). Results represent an average of three experiments. Error bars represent the s.d. of the mean. **P<0.01. (**b**) Representative immunoblots showing ROR1 knockdown at the protein level in A2780-cis cells. Top panel: ROR1; bottom panel: loading control α-tubulin. (**c**) ROR2 is decreased at the mRNA level following siRNA-induced knockdown in A2780-cis cells. No significant effect on ROR1 mRNA level. qRT–PCR was performed in triplicate and normalised to three different housekeeping genes (*SDHA*, *HSPCB* and *RPL13A*). Results represent an average of three experiments. Error bars represent the s.d. of the mean. ***P<0.001. (**d**) Representative immunoblots showing ROR2 knockdown at the protein level in A2780-cis cells. Top panel: ROR2; bottom panel: loading control α-tubulin. (**e**) ROR1 and ROR2 are decreased at the mRNA level following siRNA-induced knockdown in A2780-cis cells. qRT–PCR was performed in triplicate and normalised to three different housekeeping genes (*SDHA*, *HSPCB* and *RPL13A*). Results represent an average of three experiments. Error bars represent the s.d. of the mean. **P<0.01. (**f**) Representative immunoblots showing ROR1 and ROR2 knockdown at the protein level in A2780-cis cells. Top panel: ROR1; middle panel: ROR2; bottom panel: loading control α-tubulin.

Successful small interfering RNA (siRNA) transfection of A2780-cis significantly decreased ROR expression at the mRNA (Figures 1a, d and g, *P<*0.01 and *P<*0.001) and protein level (Figures 1b, e and f).

**Figure 2:**
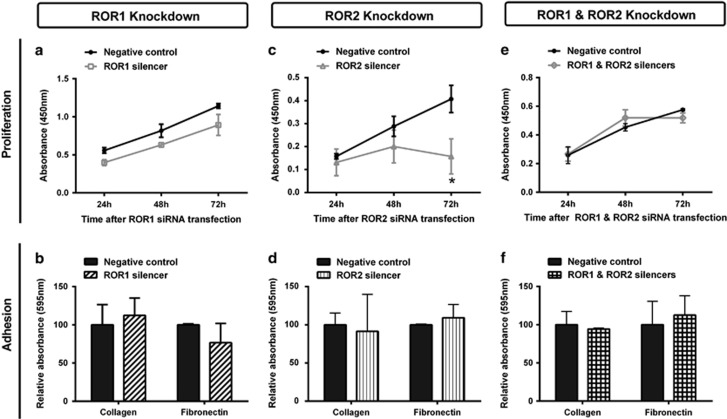
ROR1 and ROR2 silencing does not affect adhesion and may decrease proliferation. (**a**) Cell proliferation does not change over 24–72 h following ROR1 knockdown in A2780-cis cells. Results represent the average of three independent experiments. Error bars represent the s.d. of the mean. (**b**) ROR1 knockdown has no effect on the adhesion of A2780-cis cells to collagen or fibronectin. Results represent the average of three experiments. (**c**) Cell proliferation significantly decreases over 24–72 h following ROR2 knockdown in A2780-cis cells. Results represent the average of three independent experiments. Error bars represent the s.d. of the mean. (**d**) ROR2 knockdown has no effect on the adhesion of A2780-cis cells to collagen or fibronectin. Results represent the average of three experiments. (**e**) Cell proliferation does not change over 24–72 h following double ROR1 and ROR2 knockdown in A2780-cis cells. Results represent the average of three independent experiments. Error bars represent the s.d. of the mean. (**f**) Double ROR1 and ROR2 knockdown has no effect on the adhesion of A2780-cis cells to collagen or fibronectin. Results represent the average of three experiments.

ROR1 silencing had no effect on A2780-cis proliferation (Figure 2a) or adhesion (Figure 2b). However, ROR2 silencing significantly decreased cell proliferation (Figure 2c, *P<*0.05), but did not affect cell adhesion (Figure 2d). Double ROR1 and ROR2 knockdown did not have any effect on cell proliferation (Figure 2e) or adhesion (Figure 2f).

### ROR silencing in A2780-cis inhibits cell migration

**Figure 3:**
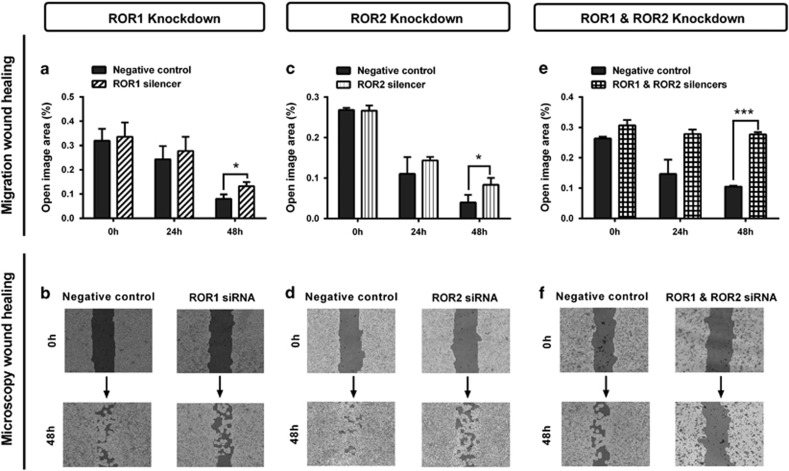
ROR1 and ROR2 silencing inhibits wound healing migration. (**a**) Wound healing cell migration is significantly decreased following ROR1 knockdown in A2780-cis cells. Results represent an average of three experiments. Error bars represent the s.d. of the mean. *P<0.05. (**b**) Representative images of wound healing analysis showing dark shading as ‘open image area' where no cells are present. Increased dark shading is seen in the ROR1 knockdown cells after 48 h incubation. (**c**) Cell migration is significantly decreased following ROR2 knockdown in A2780-cis cells. Results represent an average of three experiments. Error bars represent the s.d. of the mean. *P<0.05. (**d**) Representative images of wound healing analysis showing dark shading as ‘open image area' where no cells are present. Increased dark shading is seen in the ROR2 knockdown cells after 48 h incubation. (**e**) Cell migration is most significantly decreased following double ROR1 and ROR2 knockdown in A2780-cis cells. Results represent an average of three experiments. Error bars represent the s.d. of the mean. ***P<0.001. (**f**) Representative images of wound healing analysis showing dark shading as ‘open image area' where no cells are present. The darkest shading is seen in the double ROR1 and ROR2 knockdown cells after 48 h incubation.

**Figure 4:**
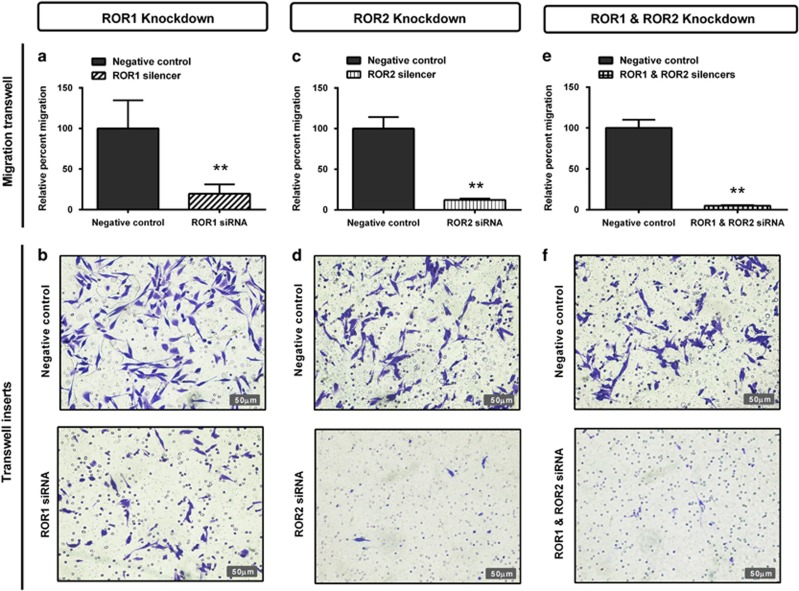
ROR1 and ROR2 silencing inhibits cell migration through transwells. (**a**) Relative cell migration performed using the transwell migration assay is significantly decreased following ROR1 knockdown in A2780-cis cells. Results represent an average of three experiments. Error bars represent the s.d. of the mean. **P<0.01. (**b**) Representative images of transwell membranes stained with crystal violet shows decreased number of cells after ROR1 knockdown. (**c**) Relative cell migration is significantly decreased following ROR2 knockdown in A2780-cis cells. Results represent an average of three experiments. Error bars represent the s.d. of the mean. **P<0.01. (**d**) Representative images of transwell membranes stained with crystal violet shows decreased number of cells after ROR2 knockdown. (**e**) Relative cell migration is most significantly decreased following double ROR1 and ROR2 knockdown in A2780-cis cells. Results represent an average of three experiments. Error bars represent the s.d. of the mean. **P<0.01. (**f**) Representative images of transwell membranes stained with crystal violet shows the strongest decrease in cell number after double ROR1 and ROR2 knockdown.

Two separate methods were used to investigate the role of RORs in cell migration: the wound healing assay (horizontal) and transwell assay (vertical). ROR1 knockdown significantly decreased wound healing migration (Figure 3a, *P<*0.05) and significantly decreased transwell migration (Figure 4a, *P<*0.01). ROR2 knockdown significantly decreased both wound healing (Figure 3c, *P<*0.05) and transwell migration (Figure 4c, *P<*0.01). Combined ROR1 and ROR2 knockdown had the strongest inhibitory effect on overall cell migration in both assays (Figures 3e and 4e, *P<*0.01).

### ROR silencing in A2780-cis cells inhibits cell invasion

**Figure 5:**
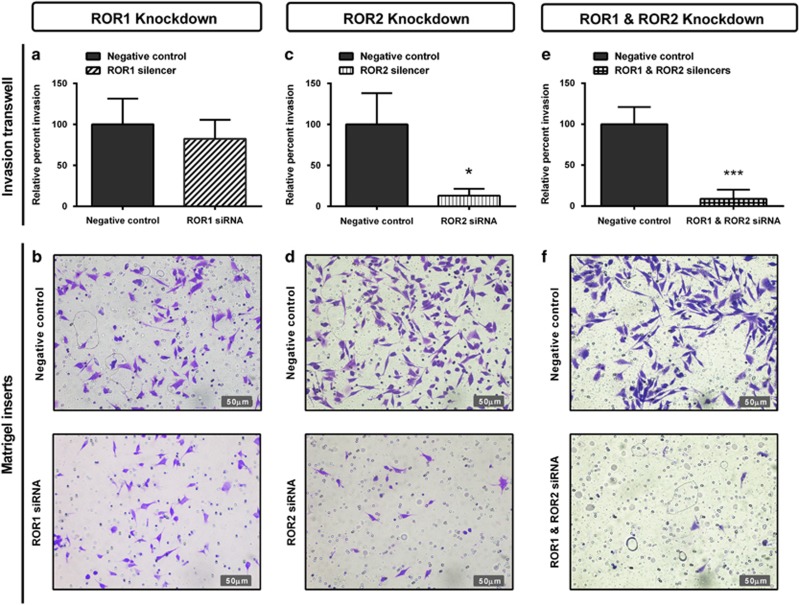
ROR1 and ROR2 silencing inhibits cell invasion through Matrigel-coated transwells. (**a**) Relative cell invasion performed using the Matrigel-coated transwell assay decreases following ROR1 knockdown in A2780-cis cells, however, is not significant. Results represent an average of three experiments. Error bars represent the s.d. of the mean. (**b**) Representative images of transwell membranes stained with crystal violet shows slight decrease in cells after ROR1 knockdown. (**c**) Relative cell invasion is significantly decreased following ROR2 knockdown in A2780-cis cells. Results represent an average of three experiments. Error bars represent the s.d. of the mean. *P<0.05. (**d**) Representative images of transwell membranes stained with crystal violet shows decreased number of cells after ROR2 knockdown. (**e**) Relative cell invasion is most significantly decreased following double ROR1 and ROR2 knockdown in A2780-cis cells. Results represent an average of three experiments. Error bars represent the s.d. of the mean. ***P<0.001. (**f**) Representative images of transwell membranes stained with crystal violet shows the strongest decrease in cell number after double ROR1 and ROR2 knockdown.

We then aimed to investigate the role of RORs in A2780-cis cell invasion through Matrigel-coated transwells. ROR1 knockdown slightly decreased invasion, although this was not significant (Figure 5a). ROR2 significantly decreased invasion (Figure 5c, *P<*0.05) and combined ROR1 and ROR2 knockdown had the strongest inhibitory effect on cell invasion (Figures 5e and f, *P<*0.001).

### Simultaneous ROR1 and ROR2 silencing sensitises A2780-cis cells to cisplatin

**Figure 6:**
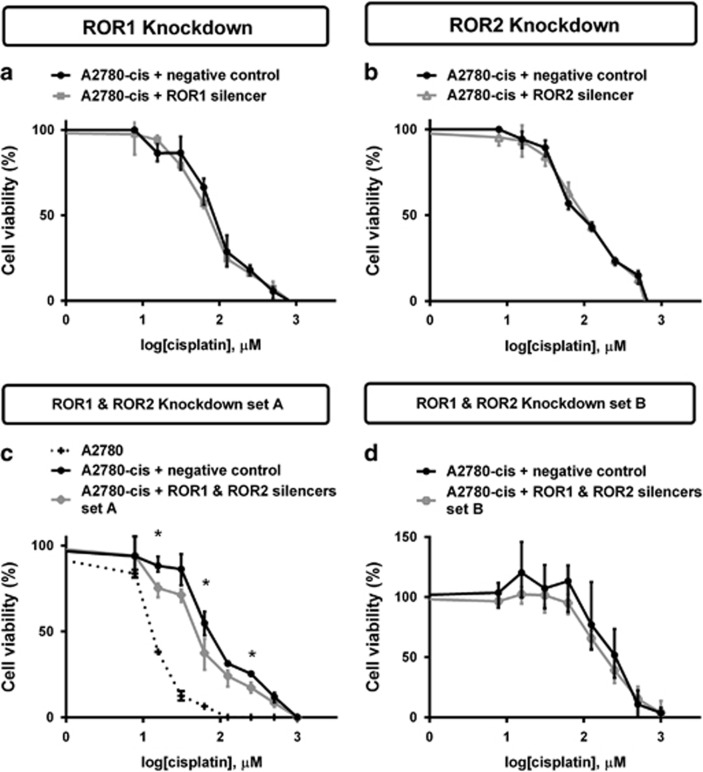
Combined ROR1 and ROR2 silencing sensitises A2780-cis cells to cisplatin. (**a**) ROR1 knockdown has no effect on cell viability when treated with cisplatin. Results represent an average of three experiments. Error bars represent the s.d. of the mean. (**b**) ROR2 knockdown has no effect on cell viability when treated with cisplatin. Results represent an average of three experiments. Error bars represent the s.d. of the mean. (**c**) Simultaneous ROR1 and ROR2 knockdown decreases A2780-cis chemoresistance and was significant at three concentration points using silencers set A. Significance was determined only between the black (negative control) and grey (ROR1- and ROR2-silenced) cells. The dotted line indicates chemosensitivity of the A2780 cell line for comparison. A2780-cis Results represent an average of three experiments. A2780 results represent an average of two experiments. Error bars represent the s.d. of the mean. *P<0.05. (**d**) Simultaneous ROR1 and ROR2 knockdown decreases A2780-cis chemoresistance using silencers set B, however, is not statistically significant. A2780-cis results represent an average of three experiments. Error bars represent the s.d. of the mean.

A2780-cis cells were treated with increased concentration of cisplatin to determine cell viability. ROR1 and ROR2 knockdown separately had no effect on chemoresistance (Figures 6a and b). However, combined ROR1 and ROR2 knockdown had a minor but statistically significant chemosensitising effect on the A2780-cis cells to cisplatin when compared with the control cells (Figure 6c, *P<*0.05). This result was replicated using an additional set of ROR silencers (Figure 6d).

### Neither ROR1 nor ROR2 overexpression affects A2780 cell proliferation or migration

**Figure 7:**
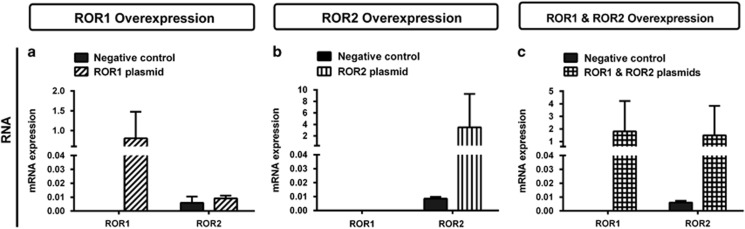
ROR1 and ROR2 overexpression in the A2780 parental cell line. (**a**) ROR1 is overexpressed at the mRNA level following ROR1 plasmid transfection. No effect seen on ROR2 levels. qRT–PCR was performed in triplicate and normalised to three different housekeeping genes (*SDHA*, *HSPCB* and *RPL13A*). Results represent an average of three experiments. Error bars represent the s.d. of the mean. (**b**) ROR2 is overexpressed at the mRNA level following ROR2 plasmid transfection. No effect seen on ROR1 levels. qRT–PCR was performed in triplicate and normalised to three different housekeeping genes (*SDHA*, *HSPCB* and RPL13A). Results represent an average of three experiments. Error bars represent the s.d. of the mean. (**c**) Simultaneous transfection of ROR1 and ROR2 plasmids in A2780 increases mRNA levels of ROR1 and ROR2. qRT–PCR was performed in triplicate and normalised to three different housekeeping genes (*SDHA*, *HSPCB* and *RPL13A*). Results represent an average of three experiments. Error bars represent the s.d. of the mean.

We then investigated the role of RORs in this model through ectopic expression in the parental A2780 cell line. Successful plasmid transfection of A2780 was confirmed by quantitative PCR with an increased ROR expression at the mRNA level (Figures 7a–c).

**Figure 8:**
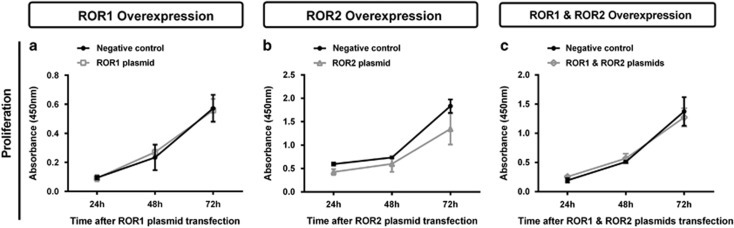
ROR overexpression has no effect on A2780 proliferation. (**a**) Cell proliferation does not change over 24-72 h following ROR1 overexpression in A2780 cells. Results represent the average of three independent experiments. Error bars represent the s.d. of the mean. (**b**) Cell proliferation does not change over 24–72 h following ROR2 overexpression in A2780 cells. Results represent the average of three independent experiments. Error bars represent the s.d. of the mean. (**c**) Cell proliferation does not change over 24–72 h following simultaneous ROR1 and ROR2 overexpression in A2780 cells. Results represent the average of three independent experiments. Error bars represent the s.d. of the mean.

**Figure 9:**
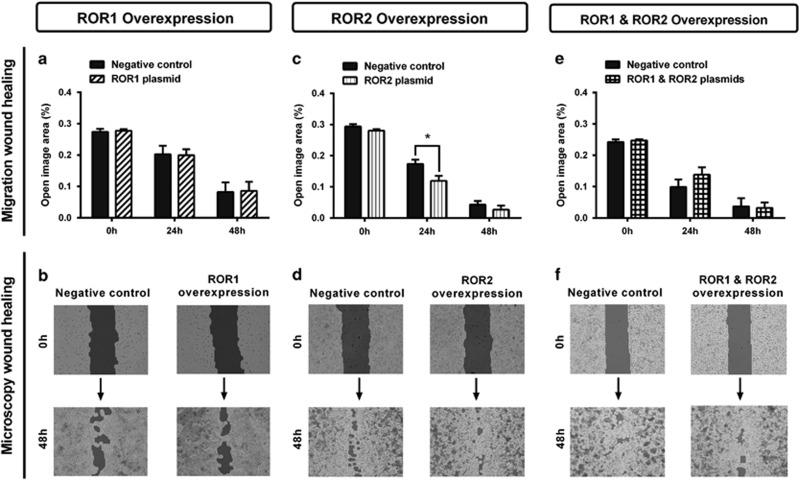
ROR2 overexpression may increase wound healing migration. (**a**) Wound healing cell migration does not change following ROR1 overexpression in A2780 cells. Results represent an average of three experiments. Error bars represent the s.d. of the mean. (**b**) Representative images of wound healing analysis showing dark shading as ‘open image area' where no cells are present. (**c**) Wound healing cell migration is significantly increased at the 24 h time point following ROR2 overexpression in A2780 cells, indicated by a decrease in ‘open image area'. Results represent an average of three experiments. Error bars represent the s.d. of the mean. *P<0.05. (**d**) Representative images of wound healing analysis showing dark shading as ‘open image area' where no cells are present. (**e**) Wound healing cell migration does not change following simultaneous ROR1 and ROR2 overexpression in A2780 cells. Results represent an average of three experiments. Error bars represent the s.d. of the mean. (**f**) Representative images of wound healing analysis showing dark shading as ‘open image area' where no cells are present.

**Figure 10:**
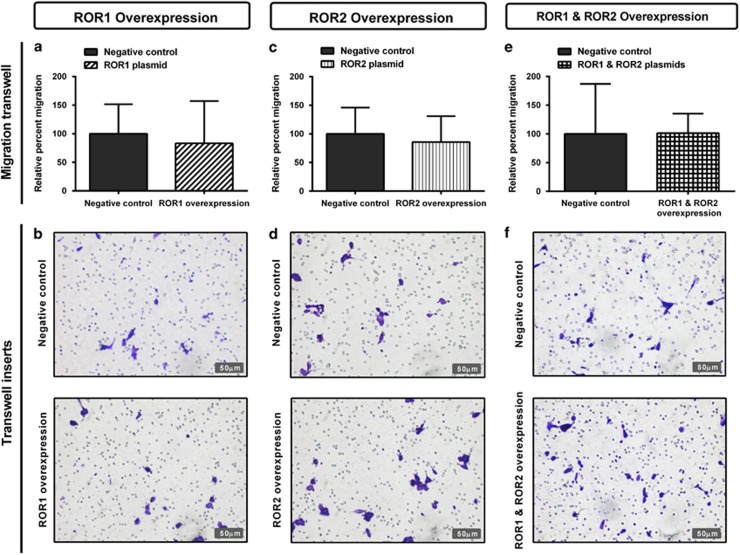
ROR overexpression has no effect on cell migration through transwells. (**a**) Relative cell migration performed using the transwell migration assay does not change after ROR1 overexpression in A2780 cells. Results represent an average of three experiments. Error bars represent the s.d. of the mean. (**b**) Representative images of transwell membranes stained with crystal violet shows similar number of cells after ROR1 overexpression. (**c**) Relative cell migration does not change after ROR2 overexpression in A2780 cells. Results represent an average of three experiments. Error bars represent the s.d. of the mean. (**d**) Representative images of transwell membranes stained with crystal violet shows similar number of cells after ROR2 overexpression. (**e**) Relative cell migration performed using the transwell migration assay does not change after simultaneous ROR1 and ROR2 overexpression in A2780 cells. Results represent an average of three experiments. Error bars represent the s.d. of the mean. (**f**) Representative images of transwell membranes stained with crystal violet shows similar number of cells after ROR1 and ROR2 overexpression.

Individual overexpression of ROR1 and ROR2, or simultaneous expression of ROR1 and ROR2 had no significant effect on cell proliferation (Figures 8a–c). There was no change in wound healing migration after ROR1 overexpression (Figures 9a and b); however, there was a significant increase in migration at the 24 h point after ROR2 transfection (Figures 9c–d, *P<*0.05). No change was seen in wound healing migration after simultaneous ROR1 and ROR2 overexpression (Figures 9e and f). Additionally, neither single ROR1 and ROR2 nor double ROR overexpression had a significant effect on cell transwell migration (Figures 10a–f).

### ROR1 overexpression increases cell invasion

**Figure 11:**
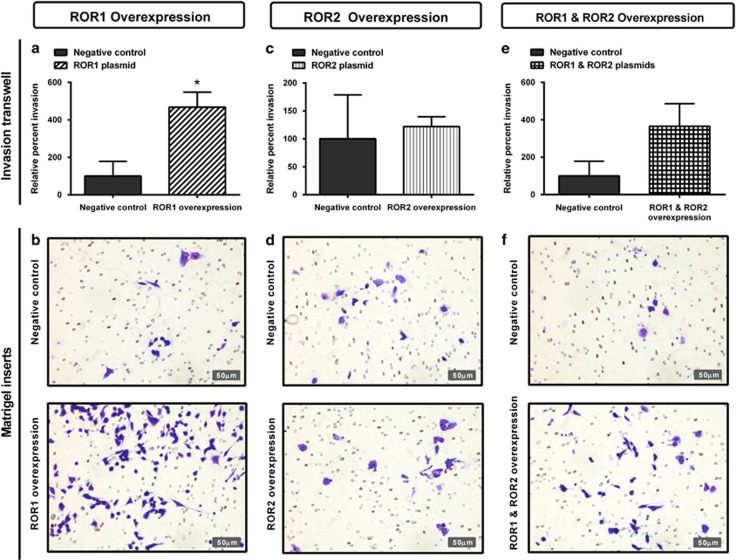
ROR1 and ROR2 overexpression increases cell invasion. (**a**) Relative cell invasion performed using the Matrigel-coated transwell assay significantly increases following ROR1 overexpression in A2780 cells. Results represent an average of two experiments. Error bars represent the s.d. of the mean. *P<0.05. (**b**) Representative images of transwell membranes stained with crystal violet shows increase in cells after ROR1 overexpression. (**c**) Relative cell invasion following ROR2 overexpression has a minor increase in A2780 cells, which is not significant. Results represent an average of two experiments. Error bars represent the s.d. of the mean. (**d**) Representative images of transwell membranes stained with crystal violet shows similar number of cells after ROR2 overexpression. (**e**) Relative cell invasion increases following double ROR1 and ROR2 overexpression in A2780 cells, however, is not statistically significant. Results represent an average of two experiments. Error bars represent the s.d. of the mean. (**f**) Representative images of transwell membranes stained with crystal violet after double ROR1 and ROR2 overexpression.

Ectopic expression of ROR1 in the A2780 cell line significantly increased cell invasion through Matrigel-coated transwells (Figures 11a and b, *P<*0.05). However, there was no change in cell invasion after ROR2 overexpression (Figures 11c and d). Simultaneous ROR1 and ROR2 overexpression resulted in an increase in cell invasion; however, this was not statistically significant (Figures 11e and f).

### ROR overexpression does not change cisplatin sensitivity in the parental A2780 cell line

**Figure 12:**
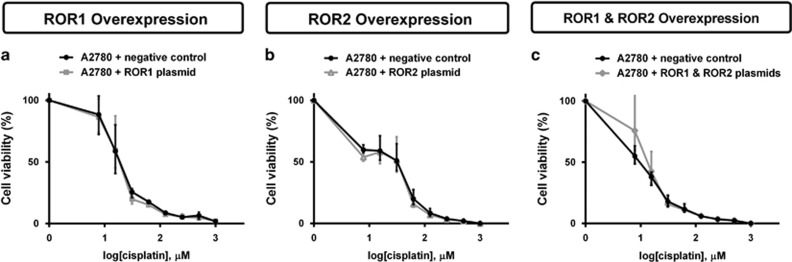
ROR1 and ROR2 overexpression has no effect on A2780 sensitivity to cisplatin. (**a**) ROR1 overexpression has no effect on cell viability when treated with cisplatin. Results represent an average of three experiments. Error bars represent the s.d. of the mean. (**b**) ROR2 overexpression has no effect on cell viability when treated with cisplatin. Results represent an average of three experiments. Error bars represent the s.d. of the mean. (**c**) Simultaneous ROR1 and ROR2 overexpression has no effect on cell viability when treated with cisplatin. Results represent an average of three experiments. Error bars represent the s.d. of the mean.

A2780 cells were treated with increased concentration of cisplatin to determine cell viability. ROR1 and ROR2 overexpression separately had no effect on chemoresistance (Figures 12a and b). Double ROR1 and ROR2 transfection was also not sufficient to significantly increase cell chemoresistance (Figure 12c).

## Discussion

Here we have shown that silencing ROR1 and ROR2 decreases cell migration and invasion in the cisplatin-resistant cell line A2780-cis. We believe this is a more clinically appropriate model for the investigation of ovarian cancer as patients often develop recurrent and resistant disease. This paper supports our previous study,^27^ which found that ROR2 was upregulated in ovarian cancer patients and that knockdown of ROR1 and ROR2 together significantly inhibited cell migration and invasion. Additionally, other groups have also reported the overexpression of ROR1 in ovarian cancer patients and linked to poor clinical outcome and increased capacity of spheroid formation.^29, 30^ Here we have additionally shown that these receptors may enhance chemoresistance, and that knockdown may sensitise these cells to cisplatin.

The ability of ovarian cancer cells to adhere to a secondary site is extremely important in ovarian cancer metastasis because of the unique dissemination process of shedding into peritoneal fluid.^31^ During the adhesion assays, we noted that the A2780-cis cell line did not adhere to collagen but only fibronectin, whereas the A2780 adhered to both extracellular matrix components. This is an interesting observation and supports the profile array results (Supplementary Figure 1E and F), suggesting that these A2780-cis cells are in a process of upregulated EMT with very low adhesion components.^12^

Recently, β-catenin-dependent Wnt signalling has been implicated in the A2780- and cisplatin-resistant cell lines.^32^ It was shown that Wnt gatekeepers such as *DKK1* and *SFRP4* were significantly downregulated in the A2780-cis cells compared with A2780. Additionally, downstream targets such as *JUN* were upregulated, supporting our current and previous results.^17, 27^ The study, however, also saw lower β-catenin protein levels in the A2780-cis cells, which may indicate some β-catenin-independent inhibitory action via the ROR receptors; unfortunately, no other β-catenin-independent Wnt targets were investigated.

Overcoming chemoresistance is a major challenge in epithelial ovarian cancer treatment and although there is initial remission in 75% of patients, subsequent recurrence occurs <2 years post-treatment.^33^ Attempts to identify characteristics of chemoresistant cells have shown markers of EMT *in vitro*.^34^ Silencing of EMT transcription factors Snail and Slug induces chemosensitivity in A2780 cisplatin-resistant cells, while upregulation leads to radiotherapy resistance and chemoresistance in A4 ovarian cancer cells.^11, 35^ In our study, we found that simultaneous silencing of ROR1 and ROR2 significantly sensitised cells to cisplatin (Figure 6c), a commonly used chemotherapy agent for ovarian cancer patients. We confirmed these results using an additional set of siRNA silencers (Figure 6d). We hypothesise that the A2780-cis cells undergo a reversion of EMT, that is, gain epithelial characteristics after double ROR knockdown, which causes their sensitivity to cisplatin. Indeed, it would be important to investigate these changes further through profiling stable knockdown and patient cell models.

During the course of the viability assays, we observed a shift in the chemoresistance of the A2780-cis cells. The viability assay with siRNA set B was completed ~4 months after set A. Therefore, it is highly likely that these cells would have changed under consistent pressure from cisplatin treatments and continual passaging. However, even though this was seen, we still noted some reduction in chemoresistance after double ROR knockdown using silencer set B (Figure 6d). The chemosensitising results, although significant at some concentration points, are minor and investigations are ongoing.

In this study, we demonstrated that ROR2 silencing alone decreased cell proliferation (Figure 2c), yet ROR1 or double ROR1 and ROR2 silencing did not (Figures 2a and e). We also found that ROR1 silencing alone had no effect on invasion (Figure 5a), yet ROR2 and double ROR1 and ROR2 silencing significantly perturbed invasion (Figures 5c and e). We also performed ROR1, ROR2 and simultaneous ROR plasmid overexpression in the parental A2780 cell line. Overexpressing the ROR receptors was not sufficient to increase migration (Figure 10) or chemoresistance of the A2780 cells (Figure 12). Interestingly, we did observe an increase in invasion after ROR1, ROR2 and double overexpression (Figure 11). ROR1 knockdown in the chemoresistant A2780-cis cell line did not significantly alter cell invasion (Figure 5a); however, ROR1 overexpression in the parental A2780 cells did. This may be because the A2780 cell line does not normally express ROR1; therefore, overexpressing the protein at very high levels could markedly change their behaviour.

These differing results between ROR1 and ROR2 after silencing and overexpression suggests that they may facilitate alternative signalling pathways as discussed previously.^27^ Recently, it has been found that ROR1 is critical in the structure and formation of caveolae^36^ and is a target for the frequently amplified *NKX2-1* gene in lung adenocarcinoma.^37^ Interestingly, its significance as a scaffold protein for a number of other RTKs was found in sustaining caveolae formation and prosurvival signalling. ROR2 may be more involved in planar cell polarity signalling through GTPases RhoA and Rac, a branch of the β-catenin-independent Wnt signalling pathway.^38^

## Conclusion

ROR1 and ROR2 are upregulated in a chemoresistant model of ovarian cancer and regulate cell migration and invasion through EMT. Targeting the ROR receptors provides a potential avenue for novel therapeutics to overcome the current inevitable development of chemoresistance in ovarian cancer patients.

## Materials and methods

### Cell culture

The epithelial ovarian cancer cell line A2780 was kindly donated by Dr Michelle Henderson (Children's Cancer Institute, UNSW, Sydney, Australia) and was originally purchased from the American Type Culture Collection (ATCC, Manassas, VA, USA). Its daughter chemoresistant cell line A2780-cis was obtained from Sigma-Aldrich (no. 93112517, St Louis, MO, USA). Both cell lines were cultured as per the supplier's recommendations (RPMI-1640 containing 10% foetal bovine serum). Media were supplemented with penicillin/streptomycin and GlutaMAX (Life Technologies, Carlsbad, CA, USA). A2780-cis cells were maintained in 1μm cisplatin every 2–3 passages. Cells were grown in 5% CO_2_ at 37 °C and were routinely tested negative for mycoplasma contamination.

### ROR silencing

Cells were transfected with siRNA targeting ROR1 and ROR2 as described previously.^27^ ROR silencing efficiency was confirmed by western blotting and quantitative real-time polymerase chain reaction (qRT–PCR) as described previously.^27^

### ROR overexpression

A plasmid encoding human ROR2 with pFLAG tagged at the N-terminal end was constructed by subcloning the ROR2 cDNA transcript into pFLAG-CMV-4 plasmid (Sigma-Aldrich). ROR1 mouse expression plasmid (pcDNA3-mRor1-flag) was generously donated by Prof Yasuhiro Minami (Kobe University, Kobe, Japan). Plasmid transfections were conducted using polyethylenimine (Polysciences, Warwrington, PA, USA) according to the manufacturer's specifications. In all transfection experiments, 1 × 10^6^ cells were seeded into 6-well plates and serum starved overnight. Cells were transfected the following day with 700 ng of ROR1 or ROR2 expression vector, or an empty vector pFLAG control (labelled in figures as negative control). Transfection mixture was removed 24 h later and cells were washed with serum-free media and replaced with complete media containing 10% foetal bovine serum. The efficiency of ROR overexpression was confirmed by western blotting and qRT–PCR. In addition to the primers previously used to detect ROR1 and ROR2,^27^ additional ROR1 primers were used to detect the ROR1 expression plasmid (F: 5′-GGGCAACCAACTATGGCTCT-3′ R: 5′-TGTTGCCACACACTGGAAGT-3′).

### EMT PCR Array

Human EMT RT^2^ Profiler PCR Array (PAS0907; SABiosciences Qiagen, Valencia, CA, USA) and RT^2^ Real-Timer SyBR Green/ROX PCR Mix (SABioscience Qiagen) was used to measure mRNA expression levels of 93 EMT genes in the A2780 and A2780-cis cell lines according to the manufacturer's protocol. Each array was repeated in triplicate.

### Proliferation assay

As described previously,^27^ cell proliferation was measured using the CCK8 Kit (Dojindo, Rockville, MD, USA) according to the manufacturer's protocol. Briefly, 7 h after ROR siRNA or plasmid transfection, 100 μl of cells were seeded in triplicate into a 96-well plate at a concentration of 4 × 10^4^ cells per ml in triplicate. At 3 h after the addition of CCK8, absorbance was read at 450 nm using the SpectraMax 190 Microplate reader (Molecular Devices, Sunnydale, CA, USA). Each assay was repeated in triplicate.

### Viability assay

Cell viability was measured using the CCK8 Kit (Dojindo) according to the manufacturer's protocol. At 24 h after ROR siRNA or plasmid transfection, 100 μl of cells were seeded in triplicate into a 96-well plate at a concentration of 4x10^4^ cells per ml. They were left to adhere for 24 h, treated with cisplatin in serial dilutions up to a concentration of 1000 mm and left to incubate at 37 °C for another 24 h. At 3 h after the addition of CCK8, absorbance was read at 450nm using the SpectraMax 190 Microplate Reader (Molecular Devices). A2780 cell viability was repeated in duplicate and used as a comparison for A2780-cis, which was repeated in triplicate. A second set of ROR silencers were used to validate the results, as described previously.^27^

### Adhesion assay

As described previously,^27^ cell adhesion was measured against collagen type I and fibronectin. After 2 h of incubation on precoated collagen or fibronectin plates, cells were stained with crystal violet and lysed with acetic acid. Absorbance was measured at 595 nm using the SpectraMax Plus190 Microplate Reader (Molecular Devices). Each assay was repeated in triplicate.

### Wound healing assay

Wound healing was analysed using IBIDI Culture-Inserts (IBIDI GmbH, Martinsried, Germany) as described previously.^27^ Cells were plated at a concentration of 1 × 10^5^ cells per ml, and after 24 h of incubation, culture inserts were removed. Photographs of the movement of cells into the scratch area were taken every 6–12 h until the scratch area had closed using a Leica DMIL microscope (Leica Microsystems, North Ryde, NSW, Australia). Wound healing was then analysed using TScratch software (ETH Zurich, Zurich, Switzerland).^39^ Each assay was repeated in triplicate.

### Transwell migration assay

Cell migration was measured using Transwell inserts (Corning Life Sciences, Tewksbury, MA, USA) as described previously,^27^ according to the manufacturer's instructions. The transwells for the A2780 cells required overnight precoating of collagen (10 μg/ml in H_2_O) before seeding. Cells were seeded in transwell inserts at a concentration of 1 × 10^6^ cells per ml (200 μl) and incubated overnight. ImageJ (Java) software (Rasband, NIH, Bethesda, MD, USA) was used to obtain an average cell count of the four stained membrane images. Each assay was repeated in triplicate.

### Transwell invasion assay

Cell invasion was measured using Matrigel precoated transwell inserts (BioCoat Matrigel Invasion Chambers, Corning Life Sciences, Tewksbury, MA, USA) according to the manufacturer's instructions as described previously^27^ and were optimised using untreated cells. Cells were seeded in transwell inserts at a concentration of 5 × 10^5^ cells per ml (100 μl) and incubated for 48 h. ImageJ (Java) software was used to obtain an average cell count of the four stained membrane images. Each ROR silencing assay was repeated in triplicate and each ROR overexpression assay was completed in duplicate.

### Statistical analysis

As described previously,^27^ all *in vitro* experimental results are expressed as mean±s.d. An F test was first used to determine equal or unequal data variance (s.d.). A Student's *t-*test type 2 was used to determine significance if equal variance. If unequal variance, a Student's *t-*test type 3 was used to determine significance. *T*-test values below *P<*0.05 were considered statistically significant. *P<0.05, **P<0.01 and ***P<0.001.
