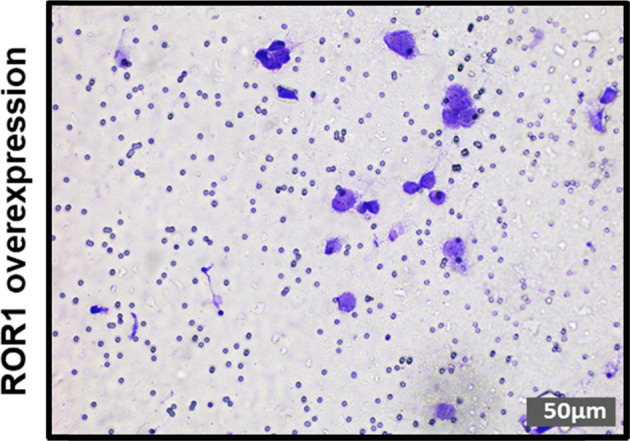# Correction to: Migration and invasion is inhibited by silencing ROR1 and ROR2 in chemoresistant ovarian cancer

**DOI:** 10.1038/s41389-022-00408-4

**Published:** 2022-06-08

**Authors:** C. E. Henry, E. Llamosas, A. Djordjevic, N. F. Hacker, C. E. Ford

**Affiliations:** 1 Adult Cancer Program, Level 2, Metastasis Research Group, Lowy Cancer Research Centre and School of Women’s and Children’s Health, Faculty of Medicine, University of New South Wales, Sydney, NSW Australia; 2 Gynaecological Cancer Centre, Royal Hospital for Women, Sydney and School of Women’s and Children’s Health, Faculty of Medicine, University of New South Wales, Sydney, NSW Australia

Correction to: *Oncogenesis* 10.1038/oncsis.2016.32, published online 30 May 2016

The authors have become aware of an error in Figure 10 and wish to issue a corrigendum for this paper.

An incorrect representative migration assay image in Figure 10b has been inadvertently added and seems to overlap with Figure 10d.

The inclusion of the incorrect image does not change the overall results, and did not contribute to the quantitation of this set of experiments.

The correct figure (Figure 10b, ROR1 overexpression) is provided below. The authors wish to apologise for this unintentional error.