# Supplementary figures and images for: Migration and invasion is inhibited by silencing ROR1 and ROR2 in chemoresistant ovarian cancer
Source: Oncogenesis. 2016 May 30;5(5):e226–. doi: 10.1038/oncsis.2016.32 (PMC4945749; doi:10.1038/oncsis.2016.32)

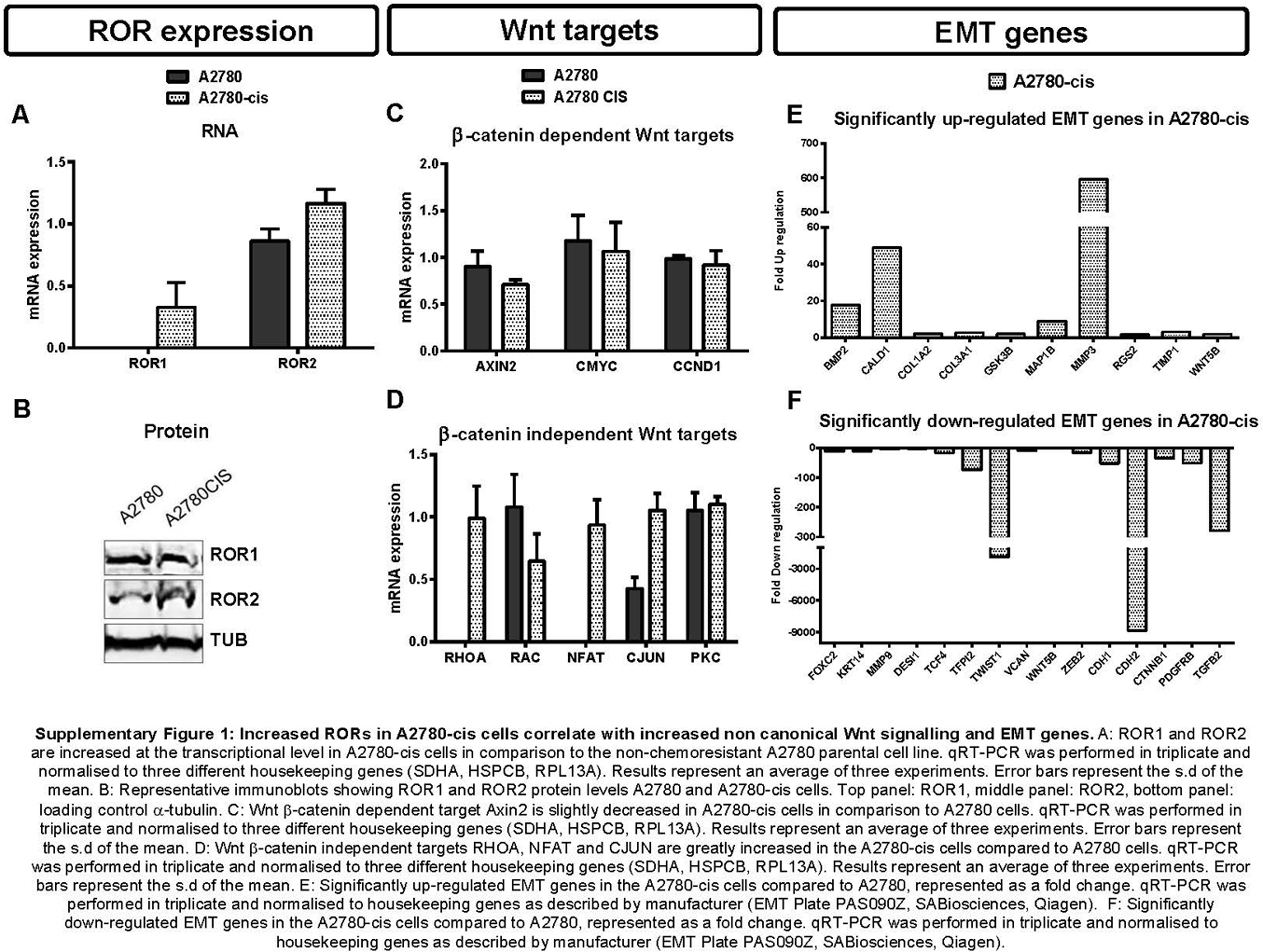

Supplement: Supplementary Figure 1 [file oncsis201632x1.tif]
